# E-cigarette use and perceived health change: Better health through vaping?

**DOI:** 10.18332/tid/95218

**Published:** 2018-10-10

**Authors:** Joy L. Hart, Kandi L. Walker, Clara G. Sears, Alexander S. Lee, Stanley Lee Ridner, Rachel J. Keith

**Affiliations:** 1 Department of Communication, University of Louisville, Louisville, United States; 2 AHA Tobacco Regulation and Addiction Center, Dallas, United States; 3 College of Nursing, East Tennessee State University, Johnson City, United States; 4 School of Medicine, University of Louisville, Louisville, United States

**Keywords:** tobacco, e-cigarette (e-cig), vape shop, electronic cigarette, vape

## Abstract

**INTRODUCTION:**

As e-cigarette use increases, questions about individual and public health effects remain unanswered (e.g. cessation tool, addiction path). Despite increasing use, few studies have focused on vape shop patrons. This study examined whether vape shop patrons believe their health is affected by the use of e-cigs; more specifically, the aim was to evaluate the association between e-cig use, change in tobacco use, and perception of health.

**METHODS:**

A survey of e-cig users (N=78) was conducted in vape shops. Questions included e-cig and traditional tobacco use, health perceptions, and demographics. Descriptive techniques were used to characterize participants as either those who perceived e-cig use improved their health or those who perceived their health unaffected. Logistic regression assessed the association between change in tobacco use, e-cig use, and perception of health effects.

**RESULTS:**

Most reported daily e-cig (91%) and current (11.5%) or former (78.2%) combustible cigarette use. Approximately, three-fourths (76.9%) perceived better health; the remainder (23.1%) perceived unaffected health. Change in cigarette use was significantly associated with perceptions that health is better with e-cig use. Participants who decreased cigarette use by 2–3 cartons/month and more than 3 cartons/month were significantly more likely to indicate that e-cig use has improved their health compared to those who decreased tobacco use by 1.5 cartons or fewer per month (OR=4.35, 95% CI: 1.13–16.9; OR=25.67, 95% CI: 2.97–221.7, respectively).

**CONCLUSIONS:**

The majority of e-cig users perceived better health. Our findings suggest that health campaign designers should carefully assess the scientific uncertainty surrounding the use of these devices and consider means to clearly convey this information. Given the lack of scientific agreement on the health effects of e-cigs and the important role that perceptions play in behavior, health campaign designers, health education practitioners, policy makers, and health care providers should err on the side of caution when advising individuals about e-cig use.

## INTRODUCTION

The advent of electronic cigarettes (e-cigs) has opened a potential new front in treatment and addiction. Some tout the devices as a harm reduction strategy that could promote smoking cessation in millions of cigarette smokers^1,2^. Others view e-cigs as a new pathway to nicotine addiction, especially for countless adolescents and young adults^3,4^. Further, concerns abound due to the potential for dual- or poly-use (i.e. using e-cigs in combination with one or more other tobacco products) as well as for the non-smoking population to be drawn to e-cigs due to marketing claims suggesting that the products do no harm. In a recent position statement^5^, the Forum of International Respiratory Societies concluded that: ‘potential benefits to an individual smoker should be weighed against harm to the population of increased social acceptability of smoking and use of nicotine’. But other groups assert that e-cigs are beneficial to public health because they provide smokers with alternatives to tobacco, thereby decreasing harmful effects^6^. Although use of e-cigs may prove helpful to some combustible cigarette smokers who are unwilling or unable to quit, or to heavy smokers seeking to decrease the number of cigarettes consumed, their overall usefulness as a cessation tool or in reducing overall harmful effects needs to be ascertained by further studies. Competing recommendations, as well as emerging scientific equivocal findings, have left health care providers, policy makers, and health education practitioners without clear guidance in formulating recommendations to current smokers and individuals interested in vaping.

Since e-cigs were introduced to the US marketplace about a decade ago, adult awareness and use of these products have been increasing. For example, recent estimates suggest that over four-fifths (83.6%) of adults were aware of e-cigs and one-fifth (22.4%) had used an e-cig at least once^7^. The perception that e-cigs have lower health risks compared to combustible cigarettes has been found also among adults^8-11^ . For example, one study reported that among current adult smokers, e-cigs were thought to have lower health risks than combustible cigarettes, snus and dissolvable forms of tobacco^10^. Another found that nearly 60% of dual users (i.e. combustible cigarettes and e-cigs) believed that nicotine replacement therapy (which is FDA approved) was as harmful as e-cigs^11^. Recent evidence, however, suggests that views of the harm perceptions of e-cigs compared to traditional tobacco products may be shifting. According to one study in 2012, over half (50.7%) of study participants viewed e-cigs as less harmful than combustible cigarettes; however, by 2014, this decreased^12^ to 43.1%. Further, some recent evidence suggests that between 2012 and 2015 more adults perceived e-cigs as posing equal or greater harm than combustible cigarettes (12.9% and 39.8%, respectively)^13^.

In line with the growing popularity of e-cigs, there has been a proliferation of vape shops, specialty retailers serving customers interested in e-cigs. Despite the increase in vape shops, relatively few studies have focused on vape shop patrons (e.g. product perceptions, use patterns). Research with vape shop customers in the US^14^ and Canada^15^ found that these e-cig users tended to be current or former smokers who used advanced equipment and viewed vaping as a means to reduce or eliminate smoking. Further, many vape shop customers perceived e-cigs as being of relatively lower harm and even beneficial to their health. A recent examination of online vape shop customers yielded similar results^16^. For example, the majority of these online purchasers reported health benefits and, although some perceived vaping as harmful, most viewed it as low in harm (i.e. either not harmful or not particularly harmful).

In this study, we explored the perceptions of e-cigs held by vape shop customers in a geographic area that historically has had favorable views and higher use rates of traditional tobacco products. We examined whether vape shop patrons believed that their health is affected by the use of e-cigs and whether such use has changed their consumption of traditional tobacco products. We also characterize the populations that perceive the most health benefits of e-cigs. The purpose of this study was to evaluate the association between e-cig use, change in tobacco use, and perception of health.

## METHODS

### Study procedures

A cross-sectional survey of e-cig users was conducted in 9 vape shops across Louisville, Kentucky, in 2015. By sampling vape shops in different areas of the city we hoped to capture a sample that reflected vape shop customers across Louisville, not just the e-cig users of one demographic and/or geographical part of the city. After approval by the University’s Institutional Review Board (IRB number 14.0493) and by the vape shop owners, the survey was administered during afternoon and evening business hours when vape shops were at their busiest. Customers were approached by a member of the research team and asked to complete the questionnaire while in the store.

### Survey methods

The questionnaire used in this survey was developed by the investigators and consisted of 39 questions about e-cig use, traditional tobacco use, perceptions of health, and demographics. Questions were adapted from the CDC National Adult Tobacco Survey (NATS) questionnaire to capture tobacco use behaviors. Vape shop customers and employees who were at least 18 years old were invited to complete the survey. Participants did not receive an incentive payment and completed the questionnaire in approximately 10 minutes while in the store.

### Measures and definitions

Perceived health effects of e-cig use were assessed by the question: ‘In your opinion, how has using e-cigarettes affected your health?’. Participants were given the response options: ‘My health is better’, ‘My health is worse’, and ‘My health has not been affected’. No participant selected ‘My health is worse’, so the variable was dichotomized.

Questions about past and current tobacco use were used to characterize participants’ perceived change in tobacco use since vaping initiation. Participants reported the number of cigarettes (all references to participant cigarette use in this study refer to combustible cigarettes) smoked per day, as well as the number of days smoked per month for two periods, corresponding to the present and prior to using e-cigs. Responses to these four questions were used to calculate the number of cigarettes smoked per month before e-cig use and after becoming an e-cigarette user. The number of cigarettes/month before being an e-cig user was subtracted from the number of cigarettes/month currently smoked to obtain the perceived change in cigarette use attributed to e-cig use.

E-cig use was characterized by questions about quantity of e-liquid (mL) used per day and number of days vaped in the past month. Participants who did not indicate the quantity of e-liquid used per day (n=8; Health better: n=3, Health not affected: n=5) were assigned the study sample median amount. All of the participants who did not report the quantity of e-liquid used per day indicated spending less than $125 per month on e-cigarettes and equipment. Based on the quantity of e-liquid used by other vape shop customers spending a similar amount (median=3 mL/day), assigning the overall median amount of e-liquid to participants with missing values is an acceptable approach. These questions were used to calculate e-liquid (mL) used per month.

### Statistical analysis

We used Fisher’s Εxact Τest and Wilcoxon Rank Sum p-values to compare categorical and continuous demographics and e-cig use behaviors between participants who perceived that e-cig use has improved their health and those who perceived that their health was not affected.

Change in cigarette use was calculated (cigarettes/month) and categorized into three groups based on the distribution of the overall sample and standard quantities of cigarettes in packages (20 cigarettes/pack and 10 packs/carton). The categories for change in cigarette use were less than 1–1.5 cartons/month, 2–3 cartons/month, and more than 3 cartons/month. Similarly, the quantity of e-liquid used was calculated (mL/month) and categorized into practical groups based on the standard quantity commonly purchased in a bottle of e-liquid (30 mL/bottle). The categories of e-cig use were less than or equal to 3 bottles/month, 4–5 bottles/month, and more than 5 bottles/month.

Logistic regression was used to assess the association between change in tobacco use, e-cig use, and perception of health effects. Odds ratios (OR) with 95% confidence intervals (CIs) are reported for crude models. SAS 9.4 (Cary, N.C.) was used for analysis.

### Sample characteristics

The overall study consisted of 80 participants, but two were excluded from the analysis due to incomplete survey data. Median age of participants (N=78) was 27.5 years (min= 18, 25th percentile=22, 75th percentile=39, max=58), 73.1% were male (n=57), 87.2% were Caucasian (n=66), and 71.8% were single (n=56). The majority of participants (69.2%) had attended at least some college (n=53), of which 41.5% obtained some type of college degree (n=22). In terms of use patterns, 7.7% of participants (n=6) used only e-cigs and had not smoked 100 cigarettes in their lifetime; 78.2% (n=61) identified as former smokers (i.e. smoked at least 100 cigarettes in their lifetime, but reported not smoking at all during the past month); 11.5% (n=9) currently use cigarettes ‘some days’ or ‘every day’, and two did not respond to these survey items. Approximately 76.9% (n=60) of participants perceived their health as better with e-cig use, whereas 23.1% (n=18) perceived their health to be unaffected by e-cig use.

The comparison of demographic characteristics between the two groups is presented in Table 1. There was no significant difference in the median age of those perceiving their health to be better (median=30 years; min=18, 25th percentile=22, 75th percentile=39, max=58) and those perceiving their health not to be affected (median=25 years; min=18, 25th percentile=20, 75th percentile=39, max=53). Significantly more males than females perceived their health as better with e-cig use (79.7% and 20.3%, respectively; p=0.04); however, the overall sample of females was small (n=20). There was no significant difference in age (p=0.61), education level (p=0.70), marital status (p=0.56), or overall perception of health (p=0.23) between the two groups.

**Table 1:** Participant Demographics, Louisville, Kentucky, USA, 2015 (N=78 )

	*Health is better (n=60 ) % (n)*	*Health not affected (n=18 ) % (n)*	*p*
**Gender**			0.04
Female	20.3 (12)	44.4 (8)	
Male	79.7 (47)	55.6 (10)	
**Education**			0.70
High school graduate or GED	31.0 (18)	27.8 (5)	
Some college	37.9 (22)	50.0 (9)	
College degree (2-year, or 4-year, or professional)	31.0 (18)	22.2 (4)	
**Race**			0.05^a^
White/Caucasian	88.1 (52)	82.4 (14)	
Black/African American	1.7 (1) 0 (0)		
Hispanic/Latino 0 (0)		11.8 (2)	
American Indian/Alaskan	1.7 (1)	5.9 (1)	
More than one race	8.5 (5) 0 (0)		
**Marital Status**			0.56^a^
Single, never married	56.9 (33)	66.7 (12)	
Single, divorced	17.2 (10)	5.6 (1)	
Married or domestic partner	25.9 (15)	27.8 (5)	
**Belief about their health**			0.23^a^
Excellent	27.6 (16)	16.7 (3)	
Good or very good	72.4 (42)	77.8 (14)	
Fair	0 (0)	5.6 (1)	

a Fisher’s exact test

## RESULTS

### Association between e-cig use and perception of health effects

The majority of participants used e-cigs ‘every day’ of the month (Table 2). Overall, the amount of e-liquid used per day ranged from 1 to 30 mL, with a median of 4 mL (IQR=5.75). In addition, 30 participants used 3 bottles or less of e-liquid per month, 24 used 4–5 bottles, and 24 used more than 5 bottles. Results from logistic regression models demonstrate that there is no significant association between quantity of e-cig use and perceived effect of e-cig use on health (comparing middle use to lowest: OR=1.54, 95% CI: 0.44–5.4; highest to lowest OR=3.0, 95% CI: 0.71–12.7).

**Table 2:** E-cigarette use behaviors, Louisville, Kentucky, USA, 2015 (N=78)

	*Health is better (n=60 ) % (n)*	*Health not affected (n=18 ) % (n)*	*Fisher’s exact p*
**Days of e-cigarette use per month**			0.34
Every day	93.3 (56)	83.3 (15)	
Less than every day	6.7 (4)	16.7 (3)	
**Amount of e-liquid used (mL/day)**			0.23^a^
Median (Range)	4.0 (1–30)	4.0 (1–18)	
Mean (SD)	7.3 (7.1)	4.7 (4.1)	
**Nicotine in e-liquid (mg/mL)**			0.42
None	6.7 (4)	16.7 (3)	
1–3	51.7 (31)	44.4 (8)	
4–11	31.7 (19)	22.2 (4)	
12–24	10.0 (6)	16.7 (3)	

Significance at the p<0.05 level.

a Wilcoxon rank-sum test p-value

### Association between change in tobacco use and perception of health effects

The majority of participants were former or current cigarette users (92.3%) with most of these participants (n=31) smoking 21 or more cigarettes per day before starting vaping. Participants who perceived their health as better with e-cigarette use were heavier smokers before starting vaping compared to participants who perceived their health as unaffected by e-cigarettes (Table 3).

**Table 3:** Tobacco use behaviors, Louisville, Kentucky, USA, 2015 (N=78)

	*Health is better (n=60 ) % (n)*	*Health not affected (n=18 ) % (n)*	*Fisher’s exact p*
**Smoked 100 traditional cigarettes or more/lifetime**			0.002
Yes	98.3 (59)	72.2 (13)	
No	1.7 (1)	27.8 (5)	
**Before you started vaping, how many days in a month did you usually smoke traditional cigarettes?**			<0.001
Every day	96.7 (58)	52.9 (9)	
Less than every day	3.3 (2)	47.1 (8)	
**Before you started vaping, how many traditional cigarettes did you typically smoke in one day?**			0.002 0.002^a^ 0.01^b^
None	1.7 (1)	29.4 (5)	
1–10	16.7 (10)	23.5 (4)	
11–20	35.0 (21)	29.4 (5)	
21 or more	46.7 (28)	17.7 (3)	
**Currently, how many days in a month do you usually use traditional cigarettes?**			0.03
Every day	1.7 (1)	17.7 (3)	
Less than every day	98.3 (58)	82.4 (13)	
**Currently, how many traditional cigarettes do you typically smoke in one day?**			0.13 0.20^a^
None	91.7 (55)	77.8 (14)	
10 or fewer cigarettes	6.7 (4)	11.1 (2)	
11–20 cigarettes	1.7 (1)	5.6 (1)	
21 or more cigarettes	0 (0)	5.6 (1)	

Significance at the p<0.05 level.

a Fisher’s exact p-value for none vs any cigarettes

b Fisher’s exact p-value for none and 1–10 cigarettes vs 11–20 cigarettes and 21 or more cigarettes.

The majority of participants indicated that they do not currently smoke any cigarettes (88.5%). The range of change in number of cigarettes used per month, after vaping compared to before vaping, was -35.5 to 0 with a median of -15.5 (IQR=17.5) cigarettes per month. No participant reported an increase in use of cigarettes since starting to use e-cigs. After use of e-cigs started, 24 participants decreased cigarette use by 1–1.5 cartons/month, 23 decreased use by 2–3 cartons/month, and 29 decreased use by more than 3 cartons/month. Change in cigarette use was significantly associated with the perception that health is better with e-cig use. Participants who decreased cigarette use by 2–3 cartons/month and more than 3 cartons/month after starting vaping were significantly more likely to indicate that e-cig use has made their health better compared to those who decreased tobacco use by 1.5 cartons or fewer per month (OR=4.35, 95% CI: 1.13–16.9; and OR=25.67, 95% CI: 2.97–221.7; respectively).

## DISCUSSION

This investigation evaluated the association between e-cig use, change in traditional tobacco use, and perception of health. The majority of participants perceived their health to be better since starting e-cig use. Previous work^17,18^ found that e-cig users report health benefits, such as better asthma regulation, easier breathing, less coughing, and more energy, while our results provide additional evidence that e-cig users perceive vaping as beneficial to their overall health.

There was no association between e-cig use behaviors or nicotine levels and perception of health benefits. However, participants who reported reductions in tobacco use since vaping were more likely to report better health. Thus, the perceived health benefits of vaping could derive from the reduction in use of traditional tobacco products, rather than from e-cig use directly. The majority of e-cig users in this study reported a reduction in cigarette use. This finding on reduction in cigarette consumption parallels results of previous studies with both in-person and online vape shop customers^14-16^.

Although the sample is small, some participants who smoked fewer cigarettes did not report changes in health in our study. The reasons shaping their views are unclear, and such factors warrant future research (which could range from perceptional and attitudinal studies to investigations of specific risks, such as cardiovascular, cancer and respiratory, or assessments of genetic differences such as in CYP2A6-deficient smokers).

Conclusions surrounding the positive and/or negative effects of e-cigs are unclear and the long-term health effects are unknown. For example, a recent systemic review of work on the health effects of e-cigs noted the frequency of contradictory or inconsistent findings as well as several methodological problems and conflicts of interest^19^ . Despite incomplete information on health effects, increasing evidence regarding the potential toxicity of the devices and liquids used in vaping is emerging. One investigation showed that inhaled vape contains 7 of 9 potentially hazardous chemicals detectable above a certain threshold and in exhaled vape 2 of these 9 remained elevated^20^. Carbonyls, a potentially toxic compound, are generated in e-cigs also^21,22^. Furthermore, studies suggest that levels of oxidants or reactive oxygen species found in e-cigs may be the same or higher than those in combustible cigarettes when these items are disposed^23^ and recent results revealed a buildup of plaque in mice, suggesting increased cardiovascular risk from e-cig vapor^24^.

The current lack of supporting evidence for both positive and negative health claims makes it difficult to fully understand the health effects of e-cigs; however, the perception that e-cigs are healthier than combustible cigarettes is often perpetuated by media, marketing, vape shops, and some public health officials^25^. E-cigs are not harmless and their long-term effects are unknown; increased use patterns in the US, in part, may be linked to the perception that these products are healthier than combustible cigarettes^26^. Increasing public understanding of the current level of scientific knowledge through health campaigns is important, as is educating frontline workers such as vape shop employees on emerging health findings to help them to better assist customers with questions about use and consequences.

### Limitations and contributions

Some limitations of our study need to be considered. The sample size was relatively small due to the low volume of customer traffic in some stores. Another limitation was that tobacco and e-cig use patterns were self-reported. Given that the study was cross-sectional and decreases in smoking cannot be verified, we cannot be certain participants actually reduced cigarette use. In addition, recall bias is possible, especially if participants desire to be labeled former smokers rather than dual users. Another limitation arises from the focus on e-cig users. Given this focus, our study cannot comment upon the experiences of other groups, such as individuals who may have perceived health problems from vaping and discontinued e-cig use and former smokers who tried vaping but do not currently use e-cigs; however, the perceptions and experiences of such samples would be interesting to explore in future research. Additionally, this study examined e-cig users in one city; thus, our findings may not be representative of e-cig users generally. Future research could compare perceived health benefits of e-cig use among dual (traditional tobacco and e-cig) users and e-cig only users that previously used traditional tobacco products.

Despite these limitations, the study has several strengths and contributes to the literature on e-cig use and perceptions. One strength is the focus on vape shop patrons. Although purchases at these specialty stores have burgeoned, few studies have focused on examining the use patterns and views of vape shop customers. However, much information can be gleaned by examining this set of e-cig users. Another strength of the study is the examination of e-cig use in a geographical area where tobacco product use has remained comparatively high and general positive perceptions of tobacco products have been resistant to change. Additionally, the research design connecting health perceptions and changes in consumption at two periods is of value. Even with these contributions, much work remains still to be done to gain a clearer picture of use patterns and health perceptions.

In summary, most of our participants believed that their health had improved since they began vaping. This research suggests that health campaign designers and health practitioners should carefully assess the scientific uncertainty surrounding the use of these devices and consider means to clearly convey this information. We found that when vaping is perceived to have reduced tobacco use, people perceive e-cigs as beneficial to their health. Future research into how perceptions impact actual use behaviors is important to understanding whether e-cigs replace traditional tobacco use, lead to dual use, or involve as many (or potentially more) harms as combustible cigarettes.

## CONCLUSIONS

Our results indicate that the majority of e-cig users perceived their health to be better since they began to vape. We note that the perceived health benefits of vaping could derive from reported reduction in tobacco use. Given the lack of scientific agreement on the health effects of e-cigs as well as the important role that perceptions play in behavior, health campaign designers, health education practitioners, policy makers, and health care providers should err on the side of caution when advising individuals about e-cig use. Additionally, vape shop employees, due to their key roles in assisting e-cig customers, are an important group to consider in future health messaging efforts.
